# Supplementary material for: Experiences and needs of patients facing incurable cancer and their relatives with informal care in psychosocial supporting centres in the Netherlands: A qualitative study
Source: PLoS One. 2026 Jan 2;21(1):e0339703. doi: 10.1371/journal.pone.0339703 (PMC12758698; doi:10.1371/journal.pone.0339703)
Supplement: S2 File — (DOCX) [file pone.0339703.s002.docx]

**Interview guide**

**Introduction**

First of all, I would like to thank you for your time and participation in this interview on support for IPSO visitors who are dealing with incurable cancer. My name is XXX and I am a junior researcher. You have received an information letter and consent form from us containing a brief explanation of the research and your rights as an interviewee. I will briefly repeat the purpose of the research.

**Purpose of the research:** The purpose of this project is to improve cooperation between informal care providers for people who are no longer getting better. Informal care means informal care and help and support from volunteers. The purpose of this interview is to gain an understanding of your experiences and needs regarding this care and support. Your experiences are therefore central to this conversation. Everything you say is therefore valid. It is really about your experience. The ultimate goal of this interview is to identify areas for improvement.

**Duration:** The interview will take a maximum of 60 minutes.

**Anonymity and confidentiality:** Everything you say during this interview will be treated confidentially. Your data will be protected and encrypted. Names you mention will be removed so that they cannot be traced back to you or the person you mention. The codes linked to your data will only be available to the IKNL research team working on this project. Information from this interview will only be used for this research. You may stop the interview at any time without giving a reason. If there are any questions you would rather not answer, you can indicate this.

**Recording:** The interview will be recorded and transcribed. You have given your written consent for this via the consent form. Thank you very much for this.

Before I start the recording, I would like to note down a few demographics.

- Could you tell me your age and place of residence?
- Gender: (male/female)
- Do you have children? Do you have a partner? Do you live alone or with someone else?
- Are you currently employed?
- What is your highest level of education?
- In what way are you affected by incurable cancer? (partner/relative/next of kin)

First, we will start with some more general questions. Then we will go into more detail about the course of your illness (or that of your relative). After that, we will discuss IPSO and your experiences with the services offered by your IPSO centre. Finally, I would like to know about your needs, i.e. what you have benefited from most or what you appreciate most about the care you are currently receiving.

I will now start the recording.

**START INTERVIEW**

| **General questions about the medical history and care needs** | |
| --- | --- |
| Medical history | Can you tell us something about your illness?  - What illness do you/your loved one have?  - How long have you/your loved one been receiving cancer-directed   treatment?  - When were you/your loved one told that you would not recover?  - Who told you this?  - How was this conversation? |
| It is known that, in addition to physical complaints, people often have many care needs in other areas, such as emotional support, help in dealing with others, or questions about the meaning of life. | |
| Care needs | With whom do you discuss your wishes and needs (physical, emotional and social)?  - With whom do you discuss what you need? |

| **IPSO center** | |
| --- | --- |
| First contact and meaning IPSO centra | First contact and expectations  - When did you first visit an IPSO centre?  - What prompted your first visit?  - What did you hope to find at the IPSO centre? |
| Awareness | How did you find out about the IPSO centre? |
| Visits | How often do you visit the IPSO centre? How many times a week or a month?  What is the main reason you visit the IPSO centre?  Do you go alone or with a family member/partner?  What activities have you done at the IPSO centre?  What do these activities mean to you? What was your reason for doing these activities?  What significance do your visits to IPSO have for you at this stage of your illness? |

| **Care needs** | |
| --- | --- |
| Care and support | Do you require specific care/assistance and support from IPSO now that you know you will not recover?  Do you attend activities at your IPSO centre that are specifically aimed at visitors dealing with incurable cancer?  How do you feel about these activities? |
| Social and practical care and support | Can you go to your IPSO centre for social care questions?   - This includes help with: - company, relationships - daily life and household - financial matters   What does the care and support for these care questions look like?  What do you like about this care/support? |
| Emotional care and support | Can you turn to your IPSO centre for emotional and psychological care? What does this look like?  *Optional to mention:*  *- emotional care and support from peers*  *- emotional care and support from informal carers*  What do you like about this care?  What could be improved in this area? |
| Spritiual care and support | Do you also visit your IPSO centre for life questions?  These may include:  - meaning and purpose in life philosophy  - dealing with life questions  What does this support look like?  What do you like about this care?  What could be improved in this area? |
| Lichamelijk | Can you also go to the IPSO centre for your physical care needs?  These may include:  - pain, fatigue, psychological complaints  What does this care/support look like?  What do you like about this care?  What could be improved in this area? |
| Missing information/support | What do you feel is lacking in the care and support provided by IPSO?  What information have you missed when it comes to support and informal care provision?  What could IPSO centres do better to ensure that their services are better aligned with your (palliative) wishes? |

| **Closure** | |
| --- | --- |
|  | We have discussed many things, for which we are very grateful. Is there anything else you would like to say or add? Anything that could help us improve the care and support IPSO provides to people with incurable cancer? |
| Wish | Thank you very much for your openness and time.  I have one last, concluding question:   - - If you could make a wish for people who will not get better, what would you wish for them in terms of help and support? |
