# Supplementary material for: Experiences and needs of patients facing incurable cancer and their relatives with informal care in psychosocial supporting centres in the Netherlands: A qualitative study
Source: PLoS One. 2026 Jan 2;21(1):e0339703. doi: 10.1371/journal.pone.0339703 (PMC12758698; doi:10.1371/journal.pone.0339703)
Supplement: Attachment — Submitted filename: PONE-D-25-30108_Review comments_20250814.docx [file pone.0339703.s003.docx]

**PONE-D-25-30108 Reviewer comments**

Thank you for the opportunity to review this paper, which I feel reports on an important topic for a group that frequently experiences underrepresentation in, and limitations/limited relevance of, informal care, particularly informal care offered via peer support. Overall, I feel this study was appropriately conducted and the paper is well written and in need only of relatively minor revisions. Please see my comments and suggestions below.

**Introduction**

Pg 3 lines 7-8; suggest that this also poses considerable challenge for health care systems in term of informal care/self-management support/social needs provision

Page 3 lines 22-29; can the authors indicate for readers who are unfamiliar, whether the IPSO centres tend to be in hospital/community/other setting, or a mixture of these?

Pg 3 line 31 – pg 4 line 4; do refs 10, 11, 12 offer any sub-analysis by cancer stage that might serve as a backdrop to expectations/set stage for the current study?

**Methods:**

Study design: I would like to see an upfront statement of philosophical underpinnings orientation of this qualitative study, to accompany the general (while necessary) statement that the design is a qualitative study.

Participants and setting: how many IPSO centres served as recruitment channels for this study?

Interview guide: page 5 line 7: states that ‘interview guide was based on concepts emerging from the literature review’ – please clarify, which literature review is this? Is this reported elsewhere/is the publication pending or is it an informal literature review undertaken for the purpose of interview guide development only?

Please also provide interview guide as appendix and refer the reader to this appendix in the body text.

Please specify who the test interview was conducted with – an academic? A consumer researcher or collaborator? A would-be/first participant (in which case, are they counted in the total number of participants reported in this study and is their data included in analysis, or not?)

Page 5 lines 30-31: discussion re data saturation; states 20 people in each group as expected to reach data saturation – clearly recruitment stopped prior to this, so it would be helpful to the reader to explain the reason for this, i.e. was this due to reaching saturation earlier than expected or due to no more consenting participants being available, or another reason?

Analysis: Please give a citation for thematic content analysis; the analysis process described does not differ from that of ‘thematic analysis’ described by Braun and Clarke and it would be helpful for the reader to understand whether you mean/used the same process, or something slightly different. If thematic analysis or similar, it is important to give more explicit information on choices made such as inductive/deductive analysis, semantic vs latent level of analysis and interpretation.

**Results:**

The note accompanying Table 1 is difficult to interpret – it would be more helpful to the reader to define what the thresholds were for low/medium/high education, e.g. does ‘low’ mean school completion only? Or did not complete secondary school? Etc.

**Discussion:**

In referring to the expressed preference to participating in peer groups specific to people with incurable cancer, the authors would do well to refer to existing literature in this space, e.g. work by Catherine Walshe in the United Kingdom on informal support and peer support for people with advanced cancer in the United Kingdom.

I feel it is also interesting that both people with incurable cancer and caregivers are included and analysed together – are there considerations around attending/participating in peer support separately/together?

Are there any specific considerations or suggestions that could be made around tailoring and access, e.g. could digital or other solutions be proposed for future iterations of services offered?

**Terminology:**

It would be helpful for consistency to use one term to describe participants’ relationship to incurable cancer; the paper uses both ‘confronted with incurable cancer’ (method: participants) and ‘dealing with incurable cancer’ (results: Table 1) which I suggest is somewhat informal for an academic paper; to apply to both people diagnosed with incurable cancer and their family members I suggest ‘affected by incurable cancer’ ‘facing incurable cancer’ or ‘with lived experience of incurable cancer’.

Page 12 line 14: ‘patients, loved ones and survivors’; it is unclear which group ‘survivors’ refers to; in particular, given that people with metastatic cancer are sometimes also referred to as ‘survivors’ (given a person is defined as a cancer survivor from diagnosis) please clarify the difference between patients and survivors.

In the discussion section (page 15 line 18), I suggest rethinking the term ‘remarkably’ as this suggests the finding was unexpected whereas I think the finding can be somewhat expected/understandable in this context. A term such as ‘notably’ may be more appropriate/ aligned with the authors’ intentions.
