# Supplementary material for: Experiences and needs of patients facing incurable cancer and their relatives with informal care in psychosocial supporting centres in the Netherlands: A qualitative study
Source: PLoS One. 2026 Jan 2;21(1):e0339703. doi: 10.1371/journal.pone.0339703 (PMC12758698; doi:10.1371/journal.pone.0339703)
Supplement: Attachment — Submitted filename: Response to reviewers.docx [file pone.0339703.s005.docx]

**Response to reviewers:**

**Reviewer #1**:

*Thank you for the opportunity to review the paper titled “Experiences and needs of patients with incurable cancer and their relatives with informal care in psychosocial supporting centres in the Netherlands: a qualitative study”. This paper describes the experiences and needs of people impacted by incurable cancer who visited an IPSO centre in The Netherlands for supportive care. The paper provides some insightful findings regarding the value and suitability of these services for this population group. Please see my detailed comments below.*

**Response:** We thank the reviewer for the thorough review and nice words regarding our manuscript.

*Abstract*

1. *Line 12, page 2 – this sentence would be better suited to your Results section.*

**Response**: We thank the reviewer for this comment and have replaced this line to the Results section.

2. *Abstract: If word count permits, please briefly elaborate on the perceived benefits of having separate peer groups for those with incurable cancer, and what aspects of the group set-ups made them inaccessible for some participants.*

**Response**: We thank the reviewer for this comment and have added the perceived benefits of a separate peer group to the results, see page 2, line 22 “They emphasized the benefit of having separate peer groups for those facing incurable cancer, including able to talk openly about end of life and a deeper sense of understanding and connection”

*Introduction:*

3. *Overall, the introduction is well-written and provides a clear and concise overview of what is currently known on this topic and justification for the aims of this study.*

**Response**: Thank you

4. *I would recommend reviewing the paragraph structure in the introduction to improve readability. Ensure each paragraph includes a clear topic and concluding or linking sentence.*

**Response**: Thank you for this comment, we have revised the paragraph structure of the introduction and have added linking sentences to it, see page 3-4

5. *Please provide a short explanation of what is meant by ‘informal caregiver’ in this study and expand on the justification as to why it is important to consider the perspectives of this population group, particularly in the context of incurable cancer.*

**Response**: Thank you for this comment, we understand the confusion. In this context, an informal caregiver is a person who provides care and support to someone with incurable cancer without being paid or professionally trained. They are often family members, friends, neighbours, or other close contacts of the person receiving care. Caregivers who work at IPSO are trained, and therefore are addressed as volunteers in our manuscript. They are important for this population as they can provide extensive psychosocial support, where the time of formal / medical caregivers is limited.

6. *Line 22, page 3 – if possible, please define the acronym ‘IPSO’. I would also suggest tweaking the wording slightly to ensure it’s clear that this organisation offers ‘volunteer-led’ support, rather than support for volunteers per se. In addition, could you elaborate further on the reach of this organisation? For example, how widely distributed are the centres? Do they offer online/telephone support in addition to in-person support? Are services accessible free of charge? What are their opening days/hours? Are services general or targeted to specific groups including those impacted by incurable cancer?)*

**Response:** Thank you for this comment, we have added additional information about the IPSO centers in the Netherlands, see page 3. “This national organization consists of 100 drop-in centers, all dedicated to providing volunteer-led support for people dealing with cancer [9]. The support offered by these IPSO include in-person support by volunteers (a listening ear, practical support), peer support, and participation in workshops (creative, physical). The volunteers are trained and supervised by a professional coordinator. Visitors report that they visit IPSO centers primarily to relax, but also to seek professional advice and meet peers [10]. IPSO centers provide a unique and accessible form of informal care that addresses both practical and emotional needs of people affected by cancer.” and page 4: “Most services are open for everyone confronted with cancer as a patient, relative or bereaved relative and not specifically targeted”

7. *Lines 1-2, page 4 – ensure consistency in the punctuation of ‘wellbeing’.*

**Response:** We have checked consistency of wellbeing and changed it all to wellbeing.

8. *Line 3, page 4 – ‘impact’ on what? Please elaborate.*

**Response:** Thank your for pointing this out. We have changed impact into societal benefits, as this study was on the benefits of IPSO for our society and our healthcare system.

9. *Lines 4, page 4 – are decimal places on these numbers necessary?*

**Response:** We agree with the reviewer and have removed the decimals.

*Methods:*

10. *The authors stated that they used the COREQ checklist for their reporting; however, I cannot see this. Could the authors please confirm if this was submitted with their manuscript?*

**Response:** We have added the COREQ checklist to the submission. Unfortunately, this did not go through at the first submission.

11. *Could the authors please elaborate on how purposive sampling was used? For example, did they purposively recruit to ensure variation in age, gender, and other demographic characteristics, or focus on people with specific experiences with the IPSO service? Otherwise, I wonder if this is more convenience sampling.*

**Response**: We agree with the reviewer that our sampling was more convenience sampling. We did try to have a variation in the role (patient and relatives), but this was marginal. We have altered this in the Method section.

12. *How many IPSO centres were contacted and then subsequently agreed to distribute recruitment flyers to their visitors?*

**Response:** All IPSO centers were contacted and received information about the project, including the recruitment flyer, but not all responded. In total, we recruited patients and relatives in 19 different IPSO centers. We have added this information to the Result section, see page 9” “A total of 31 participants were recruited from 19 different IPSO centers, ranging from 1 to 6 participants per center. The participants ‘age (18 patients and 13 relatives and bereaved relatives) ranged from 20 to 80 years old.”

13. *How was study eligibility for each visitor assessed?*

**Response**: All IPSO visitors (patients and relatives) were eligible for participation when facing incurable cancer. Incurable cancer was self-reported by the participants and not checked with their medical file. Furthermore, their age was also self-reported at the beginning of the interview. The exclusion criteria of having severe mental illness or psychological problems was not officially assessed and therefore we have removed it from the manuscript. Furthermore, we have added information about age and diagnosis to the manuscript, see page 5. “These criteria were all self-reported and not crosschecked with the medical files.”

14. *I commend the authors for collaboratively developing an interview guide based on a review of the literature and expert opinion, and for piloting this interview guide before use. I encourage the authors to include this guide in their Supplementary Materials, and to provide more detail on the following: i) what concepts were identified in the literature review and subsequently applied to the interview guide? ii) who was the interview piloted with?*

**Response:** Thank you for your kind words. We have added the interview guide to the Supplementary Materials and additional information about the literature review and pilot test, see page 5-6. “The interview guide was based on concepts emerging from the literature (including care needs on the four dimensions of palliative care, and peer support) and was developed in collaboration with an expert group from various organizations dedicated to palliative care (IPSO, Agora, VPTZ, and IKNL).”

15. *The authors have indicated that ‘data saturation’ was defined based on the description by Morse (1995). To support reader understanding, could the authors please elaborate on how this definition was applied in the context of their study?*

**Response**: Thank you for this remark. We have added additional information to the manuscript to show how we applied this in our study, see page 6: “Data saturation was based on the description by Morse [13]. Data saturation occurs when categories are theoretically rich and internally coherent. To ensure data saturation in this study, we analysed the interviews iteratively, comparing emerging themes across participants and refining conceptual categories until no new insights were observed.”

16. *Please provide a citation for the data analysis method used. It seems to reflect more of a thematic analysis approach rather than a content analysis approach.*

**Response**: Thank you for pointing this out. We totally agree with the reviewer that we used a thematic analysis approach. The word content was wrong for which we apologise and has been removed accordingly.

17. *Demographic characteristics of participants are reported. Please clarify in Methods how these data were collected.*

**Response**: We have added this information to the manuscript, see page 6 “Before the start of the interview, sociodemographic information was asked and the participants self-reported about their age, gender, marital status, level of education and working situation.”

Results:

18. *Table 1 - I am not sure I understand the footnote about level of education. Please provide further explanation. I have also not seen education categorised as ‘low’, ‘middle’, and ‘high’ before – for an international audience, please define what these represent.*

**Response:** Thank you for this comment. We have used the guidelines of the International Standard Classification of Education to define the level of education, based on the self-reported education of the participants. To make this clear, we have added to following information as footnote to Table 1: “Education was self-reported by the participants and defined according to the guidelines of the International Standard Classification of Education as low (no education, pre-primary education, primary education, lower secondary education, compulsory education, initial vocational education), medium (upper secondary general education, basic vocational education, secondary vocational education, post-secondary education) and high (specialized vocational education, university/college education, (post)doctorate and equivalent degrees).|”

19. *Line 2, page 8 – I do not think it is necessary to repeat the sample size – either report it in the Methods or Results. If possible, I encourage the authors to include a participant flow diagram showing the number of i) visitors to the IPSO centres and/or visitors approached during recruitment, ii) visitors screened as eligible, iii) visitors who consented to participate, and iv) visitors who completed an interview, to improve transparency around recruitment and sampling. As per the COREQ checklist, this should also include reasons for not participating.*

**Response**: Thank you for your feedback, we have removed the sample size from the Methods, as we believe it is more appropriate in the Results. To improve the clarity of the participants flow, we have added extra information about the number of IPSO centers, and number of participants per center included. Unfortunately, we have no information on the persons who were asked but were not interested in participating. See page 9” “A total of 31 participants were recruited from 19 different IPSO centers, ranging from 1 to 6 participants per center. The participants ‘age (18 patients and 13 relatives and bereaved relatives) ranged from 20 to 80 years old.”

20. *Line 3, page 9 – ‘IPSO hosts’ is a new term here that has not yet been introduced. Please clarify what is meant by this.*

**Response:** Thank you for your question. A IPSO host is a volunteer who has the task to welcome all visitors to the IPSO center and to provide them with coffee and tea and information. We have used the term volunteer in the manuscript, to avoid confusion.

21. *Lines 30-31, page 11 – could the authors please elaborate on this finding by explaining why some people did not perceive it necessary (or ideal) to have specific peer groups for people impacted by incurable cancer?*

**Response**: Thank you for your remark. Participants did not elaborate a lot about the specific benefits of a mixed group (curable and incurable cancer). However, this is an important nuance to the results, as not all patients expressed the need for a specific peer group as they believe it is only about end of life, and they expressed find benefits of peer support in general, see also Themes Sharing stories and experiencing recognition and a warm place with requirements.

22. *Line 21-23, page 12 – “Participants indicated that their environment sometimes draws the wrong conclusions when they talk about how they are doing, which they find frustrating” – I am a little unsure what is meant by this. Could the authors please clarify (or perhaps include an example)?*

**Response**: We have added extra information to this section, see page 13 “In addition, participants indicated that their environment sometimes misinterprets them when they talk about how they are doing. Participants express to find this frustrating.”.

23. *Line 8-9, page 13 – the second use of the word ‘practical[ly]’ is somewhat redundant in this sentence.*

**Response**: We agree with the reviewer and have removed this word, see page 14.

24. *I think it would be worthwhile including a quote to illustrate the theme of ‘a need for information’ specifically one that highlights the need for disease-specific and/or practical information.*

**Response**: Thank you for this comment. We have added some examples of need for information to the text and included a quote. This quote underlines the phase-specific information needs in general, see page 14

25. *Lines 10-11, page 14 – avoid using contraction words – e.g., ‘didn’t’.*

**Response**: Thank you for your feedback, we have altered the contraction words.

Discussion and conclusion:

26. *Overall, the authors have raised some important points regarding the suitability and value of supportive care services, such as those offered at IPSO centres, for people impacted by incurable cancer, that emerged from their findings. I recommend that the authors elaborate further on their findings regarding peer groups that are exclusive to people diagnosed with incurable cancer or to bereaved relatives. Given that not all participants supported this approach, further discussion of the perceived advantages and disadvantages would strengthen the interpretation of these findings. I also recommend that the authors compare their findings to previous research conducted with IPSO visitors, to demonstrate the novelty and uniqueness of their findings for people impacted by incurable cancer.*

**Response**: Thank you for this feedback. We have altered the discussion accordingly and believe that the urgency of the need for exclusive peer groups for patients with incurable cancer is better integrated into the Discussion section. See page 16-17 “The participants also reported the added value of dedicated peer groups for patients or relatives facing incurable cancer, as they felt able to talk openly about end of life and experienced a deeper sense of understanding and connection. However, the volatility and unpredictability of the disease makes that they cannot always visit IPSO when they have a need to and hampers the practical aspects of organizing dedicated peer groups.”

27. *Line 25, page 15 – please check the punctuation here. There is an inverted comma at the beginning of a sentence.*

**Response**: Thank you, we have altered it accordingly.

28. *Line 30, page 15 – remove ‘that’.*

**Response**: That has been removed for this sentence.

29. *For improved flow, I would suggest including the ‘Implications and further research’ section above ‘Strengths and limitations’*

**Response**: Thank you for this suggestion, we have added a paragraph on the implications and future research to the Discussion section, see page 16-17: “Implications and future research

This study highlights the importance of tailoring psychosocial support for patients and families facing incurable cancer. The findings underscore the need for dedicated peer groups that address the unique emotional and existential challenges, as mixed groups with curable cancer patients may inadvertently hinder open dialogue. Given the increasing uncertainty and variability in incurable cancer trajectories [16], Future research should explore flexible and accessible models of support that respond to evolving needs and symptom burdens, while also reflecting the cultural diversity of participants. While many current visitors have a Western European background, it is important to examine how IPSO can be made more accessible and culturally appropriate for individuals from non-Western European backgrounds.". Additionally, investigating digital or hybrid formats for peer support may offer promising avenues to overcome practical barriers related to physical attendance and timing. “

Strengths and limitations:

30. *To clarify, were all participants returning visitors as opposed to once-off visitors? If so, this should be clarified in Methods.*

**Response**: Thank you for your question. We have not recorded this of the participants, but we know from the volunteers and the IPSO coordinator that the vast majority were returning visitors. We have included this feature of the IPSO visitors in general to the description of the service of IPSO, see page 4: “The study by Sinzer [11] showed that the vast majority of IPSO visitors experience a positive change on their wellbeing, particularly in relation to their mental wellbeing and social participation. Moreover, visitors often come multiple times to IPSO, one third comes once a month and one third visits IPSO longer than 3 years [11]”.

31. *Another limitation could be a lack of contextual information for individual participants that could have an impact on their experience with the service (e.g., race/ethnicity, geographic remoteness, cancer type, duration and nature of engagement with services in the IPOC centres).*

**Response**: We agree with the reviewer that more contextual information of the participants would have been interesting for a deeper understanding of their experiences. However, the fact that we recruited patients and relatives facing incurable cancer was already a big step for some centers. Furthermore, it is known that the visitors of IPSO have dominantly a West-European background (94%), and remoteness is not really an issue in a dense populated country as the Netherlands. Unfortunately, we have no additional information to add to our manuscript. We have added the need for more research to the experiences of IPSO visitors with non-western backgrounds to the future research paragraph in the Discussion section.

32. *Implications: Lines 13-19, page 16 – perhaps provide some specific examples of how these recommendations could be achieved.*

**Response**: Thank you for this suggestion. We have added more detail to the implication, see page 16: “*The findings underscore the need for dedicated peer groups that address the unique emotional and existential challenges, as mixed groups with curable cancer patients may inadvertently hinder open dialogue. . Finally, for volunteers, it is important to be well informed about the needs and experiences of visitors facing incurable cancer, including advance care planning, end-of-life care, and bereavement. A key recommendation for future research is to investigate how many individuals living with incurable cancer may need informal care but are unable to access it due to their substantial burden of illness. It is important to explore the extent to which these illness-related limitations affect access to informal support in order to develop appropriate tailored care for this group. Future research should also explore flexible and accessible models of support that respond to evolving needs and symptom burdens, while also reflecting the cultural diversity of participants. While many current visitors have a Western European background, it is important to examine how IPSO can be made more accessible and culturally appropriate for individuals from non-Western European backgrounds.". Additionally, investigating digital or hybrid formats for peer support may offer promising avenues to overcome practical barriers related to physical attendance and timing.*”

33. *Conclusion: Line 6, page 17 – Please soften the language of “[needs]…will be addressed through peer contact”. The findings of this study allude to the fact that special peer groups may be of benefit to some people impacted by incurable cancer, but we cannot say for sure that these groups will address all the aforementioned needs.*

**Response**: Thank you for pointing this out. We have changes will into may, to soften the conclusion, in line with the qualitative results.

**Reviewer #2: Data availability:**

1. *I completely understand that raw qualitative data is not appropriate to be made available publicly due to ethical/confidentiality concerns; I simply feel this needs to be stated more clearly in the submission.*

**Response**: We would like to thank the reviewer for this statement. We agree that data availability is essential, however, we have no consent to publish the raw data (interview transcripts) publicly. We have added this in our statement.

*Thank you for the opportunity to review this paper, which I feel reports on an important topic for a group that frequently experiences underrepresentation in, and limitations/limited relevance of, informal care, particularly informal care offered via peer support. Overall, I feel this study was appropriately conducted and the paper is well written and in need only of relatively minor revisions. Please see my comments and suggestions below.*

**Response**: Thank you for your thorough review of our manuscript and your valuable suggestions to further improve it.

Introduction

1. *Pg 3 lines 7-8; suggest that this also poses considerable challenge for health care systems in term of informal care/self-management support/social needs provision*

**Response**: Thank you for this suggestion. We have added the challenge for the health care system to the Introduction, see page 3: “This growing number of patients with metastatic cancer poses a considerable challenge for patients and their relatives [2-3] and for health care systems, underlying the essential role of informal care and self-management support. These developments highlights the growing societal and healthcare burden associated with cancer, particularly metastatic disease.”

2. *Page 3 lines 22-29; can the authors indicate for readers who are unfamiliar, whether the IPSO centres tend to be in hospital/community/other setting, or a mixture of these?*

**Response**: Thank you for this question, we have added additional information regarding IPSO in order to improve the clarity about this service. See page 3: “These centers are independently located or sometimes situated near hospitals. They offer free, non-medical support provided by trained volunteers and professionals, aiming to improve quality of life and reduce isolation during and after cancer treatment.”

3. *Pg 3 line 31 – pg 4 line 4; do refs 10, 11, 12 offer any sub-analysis by cancer stage that might serve as a backdrop to expectations/set stage for the current study?*

**Response:** Thank you for this question, unfortunately in the evaluation of the IPSO services as described in these references were no sub-analyses by cancer stage performed.

Methods:

4. *Study design: I would like to see an upfront statement of philosophical underpinnings orientation of this qualitative study, to accompany the general (while necessary) statement that the design is a qualitative study.*

**Response:** We have added the statement about the study design in the Methods, together with the orientation, we believe this addition strengthens the transparency and methodological rigor of our study, see page 3: “A qualitative study grounded in a constructivist paradigm using semi-structured interviews was conducted to explore how IPSO visitors facing incurable cancer construct meaning from their experiences within the IPSO centers.”

5. *Participants and setting: how many IPSO centres served as recruitment channels for this study?*

**Response:** In total, we recruited patients and relatives in 19 different IPSO centers. We have added this information to the Result section, see page 9” “A total of 31 participants were recruited from 19 different IPSO centers, ranging from 1 to 6 participants per center. The participants ‘age (18 patients and 13 relatives and bereaved relatives) ranged from 20 to 80 years old.”

6. *Interview guide: page 5 line 7: states that ‘interview guide was based on concepts emerging from the literature review’ – please clarify, which literature review is this? Is this reported elsewhere/is the publication pending or is it an informal literature review undertaken for the purpose of interview guide development only?*

**Response:** Thank you for this question. We did not conduct a formal literature review, but used literature to develop the interview guide. To avoid this confusion, as also experienced by reviewer 1, we have deleted the word review from this sentence as we did use the literature, but not in a systematic manner, see page 6: “The interview guide was based on concepts emerging from the literature (including care needs on the four dimensions of palliative care, and peer support) and was developed in collaboration with an expert group from various organizations dedicated to palliative care.”

7. *Please also provide interview guide as appendix and refer the reader to this appendix in the body text.*

**Response:** Thank you for this suggestion. We have added the translated interview guide to the Supplementary Materials, as referred at page 6.

8. *Please specify who the test interview was conducted with – an academic? A consumer researcher or collaborator? A would-be/first participant (in which case, are they counted in the total number of participants reported in this study and is their data included in analysis, or not?)*

**Response:** The pilot or test interview was conducted with an academic, with a senior researcher with ample expertise in qualitative research. To make this clear, we have added this information to the Method section, see page 6 “A test interview was performed by JvK to evaluate the interview guide with a senior researcher with expertise in qualitative research in order to test the flow of the interview guide and to give feedback to JvK on her interview skills.”

9. *Page 5 lines 30-31: discussion re data saturation; states 20 people in each group as expected to reach data saturation – clearly recruitment stopped prior to this, so it would be helpful to the reader to explain the reason for this, i.e. was this due to reaching saturation earlier than expected or due to no more consenting participants being available, or another reason?*

**Response:** Data saturation was expected to happen after 20 people per group, but occurred earlier. This 20 was an estimation, and true data saturation occurs based on the data. Especially in the IPSO visitors – relatives, after analysing the first 10 interviews no new themes occurred. To ensure the data saturation, we have recruited some extra relatives to be sure. We have incorporated this approach into the Method section, see page 6-7: “Data saturation occurs when categories are theoretically rich and internally coherent. To ensure data saturation in this study, we analyzed the interviews iteratively, comparing emerging themes across participants and refining conceptual categories until no new insights were observed.”

10. *Analysis: Please give a citation for thematic content analysis; the analysis process described does not differ from that of ‘thematic analysis’ described by Braun and Clarke and it would be helpful for the reader to understand whether you mean/used the same process, or something slightly different. If thematic analysis or similar, it is important to give more explicit information on choices made such as inductive/deductive analysis, semantic vs latent level of analysis and interpretation.*

Response: Thank you for your comment. We totally agree with the reviewer that we used a thematic analysis approach. The word content was wrong for which we apologise and has been removed accordingly. Reviewer 1 also brought this to our attention.
 Moreover, we have made the steps of the thematic analysis more clear by adding the following information, see page 7 “All interviews were coded through thematic analysis, a qualitative analysis technique suitable for exploring themes and patterns within the data. We followed the following 6 steps: (1) familiarization with the data, (2) generating initial codes, (3) searching for themes, (4) reviewing themes, and (5) defining and naming themes, and (6) producing the report. The analysis process aimed to identify recurring themes and key patterns related to the research objectives. We conducted an inductive analysis, allowing themes to emerge from the data rather than applying a pre-existing coding frame. Furthermore, we focused on the semantic level of analysis, interpreting the explicit content of the interviews.”

*Results:*

11. *The note accompanying Table 1 is difficult to interpret – it would be more helpful to the reader to define what the thresholds were for low/medium/high education, e.g. does ‘low’ mean school completion only? Or did not complete secondary school? Etc.*

**Response:** Thank you for this comment. We have used the definition of level of education of the International Standard Classification of Education (ISCE). We have added this definition of low/medium/high level of education to the footnote of the Table and have also mentioned this into the Method section, see page 8 (Table 1) and page 6.

*Discussion:*

12. *In referring to the expressed preference to participating in peer groups specific to people with incurable cancer, the authors would do well to refer to existing literature in this space, e.g. work by Catherine Walshe in the United Kingdom on informal support and peer support for people with advanced cancer in the United Kingdom.*

**Response:** We agree with the reviewer that our manuscript would be strengthened with additional international research, thank you for pointing this research of Catharine Walshe to us. We have added her research into the Discussion section, see page 17: “Research on peer support for patients with incurable or advanced cancer is scarce, as shown by Walshe et al in a scoping review [15]. However, a recent feasibility study in the UK showed promising results of dedicated peer support for patients with advanced cancer. The recruitment of peer mentors (with advanced cancer) was unproblematic, the recruitment of participants was challenging, and this feasibility study showed promising results, indicating a positive effect on QoL, although caution is needed due to low power [16] align with visitors’ need to be understood and are mutually reinforcing.**“**

13. *I feel it is also interesting that both people with incurable cancer and caregivers are included and analysed together – are there considerations around attending/participating in peer support separately/together?*

**Response:** Thank you for this interesting thought. In the interviews, none of the participants mentioned the need of combined peer groups for patients and relatives facing incurable cancer. As one of the main drivers of participants to appreciate dedicated peer support groups is the room to express their feelings without having to worry to upset others and to experience a deeper sense of connection, as the peers have the same experience, our assumption it that such a mixed support group would to fit the need of patients and relatives. As this was not a topic that arose from the interviews, we have decided not to include this perspective into our manuscript.

14. *Are there any specific considerations or suggestions that could be made around tailoring and access, e.g. could digital or other solutions be proposed for future iterations of services offered?*

**Response:** *We agree with the reviewer that digital services and solutions may be of added value to the organisation of dedicated peer support for patients with advanced cancer. However, none of the participants mentioned this need or suggested this as potentially solution for some of the practical challenges. As none of the participants did mention this, we have not added this to the results. We did include it in the Discussion section; clinical implications and future research, as we agree that this is an interesting solution, see page 18. “*Additionally, investigating digital or hybrid formats for peer support may offer promising avenues to overcome practical barriers related to physical attendance and timing.”

*Terminology:*

15. *It would be helpful for consistency to use one term to describe participants’ relationship to incurable cancer; the paper uses both ‘confronted with incurable cancer’ (method: participants) and ‘dealing with incurable cancer’ (results: Table 1) which I suggest is somewhat informal for an academic paper; to apply to both people diagnosed with incurable cancer and their family members I suggest ‘affected by incurable cancer’ ‘facing incurable cancer’ or ‘with lived experience of incurable cancer’.*

**Response:** *Thank you for this suggestion, we have changed in throughout the manuscript into facing incurable cancer, to improve the readability of our manuscript.*

16. *Page 12 line 14: ‘patients, loved ones and survivors’; it is unclear which group ‘survivors’ refers to; in particular, given that people with metastatic cancer are sometimes also referred to as ‘survivors’ (given a person is defined as a cancer survivor from diagnosis) please clarify the difference between patients and survivors.*

**Response:** *We agree with the reviewer that it is unclear which groups survivors is referred to. Some of our participants were patients facing incurable cancer, but also felt a survivor, because the tumour activity was diminished due to immunotherapy. Therefore we used the term survivor in the manuscript. But we see the confusion and have changed it to “patients and relatives”. See page 13: “*The experiences of patients and relatives are, again, not described separately, but are combined together.”

17. *In the discussion section (page 15 line 18), I suggest rethinking the term ‘remarkably’ as this suggests the finding was unexpected whereas I think the finding can be somewhat expected/understandable in this context. A term such as ‘notably’ may be more appropriate/ aligned with the authors’ intentions.*

**Response:** We agree with the reviewer and have changed remarkably into notably.

**Academic editor**

1. *Dear Authors, could you please address the feedback suggested by the two reviewers. Additionally, in the abstract it states ‘in order to realise an appropriate fit at IPSO for this group’ is unclear. Could this please be amended? Perhaps ‘to optimise the services provided by IPSO centers’ as per the introduction.*

**Response:** Thank you for your feedback. We have amended the abstract accordingly.

2. *Additionally, the COREQ guidelines are missing for this submission as well as the interview guide as supplementary material*

**Response:** We have added the COREQ guidelines to the submission, and have added the interview guide as supplementary material to the manuscript.
